# A tough and sustainable fiber-forming material from lignin and waste poly(ethylene terephthalate)†‡

**DOI:** 10.1039/c9ra07052d

**Published:** 2019-10-01

**Authors:** Kokouvi M. Akato, Ngoc A. Nguyen, Kalavathy Rajan, David P. Harper, Amit K. Naskar

**Affiliations:** Center for Renewable Carbon, University of Tennessee Knoxville Tennessee 37996 USA naskarak@ornl.gov; Bredesen Center for Interdisciplinary Research and Graduate Education, University of Tennessee Knoxville Tennessee 37996 USA; Carbon and Composites Group, Chemical Sciences Division, Oak Ridge National Laboratory Oak Ridge Tennessee 37831 USA

## Abstract

In this report we describe repurposing of recycled polyesters as a matrix for lignin-a biorefinery coproduct that is used as a solid fuel and needs to find higher value-to make sustainable high-performance thermoplastic materials. Brittle lignin oligomers, isolated from plant biomass, require a low-melting host polymer matrix to form a rigid and tough renewable material. We demonstrate controlled lignin dispersion and interfacial interactions in softened recycled polyethylene terephthalate (PET) using a simple solvent-free, melt-blending technique. To avoid lignin degradation and devolatilization during melt processing, it was thermally treated. Tall oil fatty acid was used to enable PET processability at low enough temperature to accommodate lignin without charring. Chemical analysis reveals reduction of aliphatic hydroxyl content from 2 mmol g^−1^ to 1.63 mmol g^−1^ and an increase of total phenolic hydroxyl moieties from 5.86 to 6.64 mmol g^−1^ and cleavage of β-O-4 ether linkages due to thermal treatment. Structural transformation of lignin macromolecules during heat treatment was further confirmed by an increase in molar mass and improved thermal stability. Interfacial interactions between lignin and PET were assessed from mechanical properties and thermal analyses. Thermal treatment not only helps to improve the stability of lignin but also slightly reduces the size of the dispersed lignin domains *via* favored interfacial interactions with the PET matrix. These methods improve mechanical properties of the material. Further, incorporation of lignin in the plasticized PET matrix increases the ductility in the blended products. The method we discuss here utilizes industrial wastes and co-products, and it does not require solvent or toxic chemicals during the reactive extrusion process that yields complete conversion to products.

## Abstract 2

This study reports a path for recycling polyester along with biorefinery coproduct, lignin, to make sustainable high-performance thermoplastic materials.

## Graphical abstract

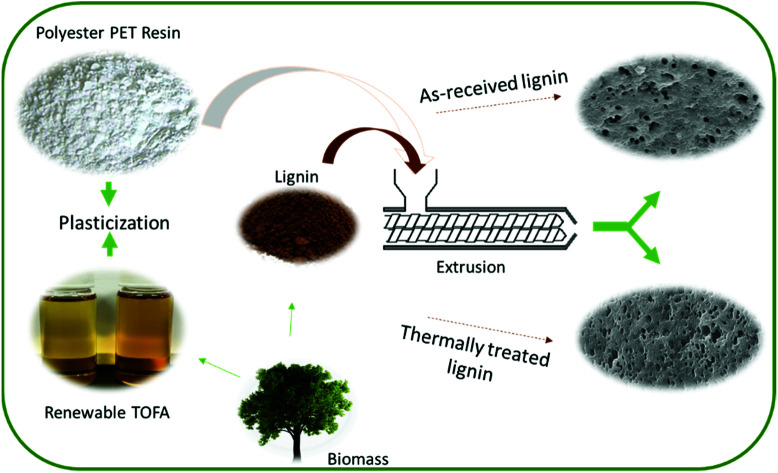

## Introduction

Conservation of petrochemicals and utilization of wastes and renewable materials are essential to avoid industrial pollution.^1^ Proposed reliable solutions include usage of materials made from renewable sources and development of value-added products from wastes.^2^ Lignin, a natural polymer in biomass, is a coproduct from paper mills and biorefineries and is a promising feedstock for renewable plastics. Both lignin utilization and recycling of waste plastic remain immature, and the technological deficiency is primarily caused by the excessive emphasis on direct use of these materials as fillers and the approach results in downgraded products. In this context we propose combination of post-consumer polyethylene terephthalate (PET) waste and lignin-currently considered as a cheap source of energy *via* combustion-to produce a new class of sustainable polymeric materials. This creates a favorable solution *via* value-addition to both renewable wastes, spent and recycled materials, and curtailing environmental concerns. However, reused PET products are very brittle, and they become further brittle when melt-mixed with lignin at the melting temperature of PET. Lignin degrades and forms char at the melting temperature of PET.

PET is a semi-crystalline thermoplastic polyester broadly used in packaging industries. Wastes generated during manufacturing and consumption of PET are detrimental to the environment because PET lacks biodegradability.^3^ Thus, recycling these wastes is desired for environmental protection, conservation of petrochemicals and energy, and to generate additional revenue streams. Currently, recycled waste PET is used in construction, packaging, and composite applications. For example, recycled PET is used readily in structural concrete reinforcement, where crack control and enhancement of ductility are valuable.^4,5^ Waste PET is also used to develop new materials through blending with other polymers.^6–9^ Most blends are immiscible at molecular levels due to unfavorable interfacial tension between the components and often require the addition of a compatibilizer.

Blends of PET and lignin were evaluated as an alternate route in the efforts of lignin valorization to derive thermoplastic/lignin alloys.^10–16^ Unfortunately, only moderate interactions between thermally degraded lignin and the PET matrix and a downward trend of the tensile strength as a function of the amount of lignin loading were observed. For example, a blend of PET and 30 wt% lignin-prepared in our laboratory at 260 °C-shows that the blend only retains less than 38% of tensile stress of waste PET and a significant decrease in elongation. Therefore, in most cases, lignin was modified to introduce functional moieties that favor strong interactions between the components. Adequately tailored interfacial interactions, either by chemical route or by addition of compatibilizer, drastically improve the dispersion of fine homogenous lignin domains that is valuable for performance enhancement of the blends. In that regard, esterification is often used as a lignin modification route.^17,18^ The additional step increases cost and creates a need for chemical disposal, which constitutes an inherent disadvantage for the new polyester-lignin blends for industrial adoption.

Most of the earlier reports on PET-lignin blends claim partial miscibility or total immiscibility between lignin and the host matrix with widespread variations in structures and properties based on microscopy, differential scanning calorimetry (DSC), and Fourier transformed infrared (FTIR) spectroscopy. Yet very little information is available on process engineering of the blends. Due to the high melting temperature (∼260 °C) of PET, choosing appropriate blending temperatures for PET/lignin blends is important to avoid lignin degradation during mechanical blending. Jeong *et al.*^14^ reported mixing temperature of 170 °C for blends of lignin and synthetic polymers including PET, which yielded heterogeneous blends of PET. Temperature setup as high as 265 °C were used elsewhere.^10^ In general, the rheology of the blend components impacts processing and phase behavior of the resulting PET/lignin blends.

The scope of this study lies within usage of melt-based blending techniques to develop partially renewable polymer blends of post-manufacturing PET waste and an organosolv lignin, a low-cost natural polymer obtained from biomass processing industries without chemical modification. In general, normal processing temperatures (265 °C to 280 °C) of post-manufacturing PET are deemed detrimental for nanoscale dispersion of lignin without thermal degradation of the lignin phase. For this reason, lignin can be thermally treated to remove low molecular weight volatile materials and improve its heat resistance during blending. It also helps to avoid devolatilization that negatively impacts the blend morphology (by creating porosity) and properties. Based on our previous report,^19^ a renewable plasticizer-tall oil fatty acid (TOFA) that is essentially oleic acid enriched oil-at 10 wt% relative to PET was added to help soften PET chains and to reduce its melt-processing temperatures by 20 °C. TOFA is another co-product (apart from lignin) of the paper industry, and plant-derivatives used here in combination with the recycled PET results in lignin-based thermoplastic alloys that are malleable and reprocessable. The process does not need any solvent or toxic chemicals to make products. The process *via* melt-phase extrusion does not generate significant wastes. Thus, it fulfills basic principles of green chemistry. Combining thermal treatment with plasticizing permits appropriate choices in mixing temperature, to control dispersion of lignin, and associated promotion of interfacial interaction that are necessary to create higher performance sustainable composites of lignin. In summary, this study involves adept characterization of lignin, its structural transformation during thermal treatment followed by an assessment of interfacial interactions of lignin in PET matrix, and subsequent correlation to morphology and mechanical properties of sustainable PET/lignin blends.

## Experimental section

### Materials

Thermoplastic polyester PET was received from Eastman Chemical USA. It is scrap from the resin manufacturing facility and supplied as white ground plastic. Melt flow rate (MFR) measured in our laboratory is 55 g/10 min at 280 °C at 2.16 kg applied force. The PET was dried under vacuum at 130 °C for 12 hours to avoid hydrolytic degradation during melt processing. Organosolv hardwood lignin (L) was provided by Lignol Innovations, Canada. The lignin melts fully at 147 °C and flows at 163 °C (Fisher Scientific melting point tester). The lignin was dried at 60 °C for 8 hours. The tall oil fatty acid (TOFA) was acquired from Westvaco Chemicals, Charleston SC. It is a Westvaco L-5 Tall oil fatty acid that primarily consists of oleic acid. The specifications of the TOFA were reported as: acid number (min 190), rosin acids (max 5%) and color or Gardner (max 7).

### Lignin thermal treatment and characterization

The lignin (L) was thermally treated in a vacuum oven at 200 °C for 60 minutes to improve its thermal stability. The thermally treated lignin is identified as L_HT_. Both as-received L and L_HT_, were characterized using Gel permeation chromatography (GPC) to evaluate molecular weight and molecular weight distribution (see ESI‡). Functional features were characterized and quantified by ^31^P NMR and 2D ^1^H–^13^C HSQC NMR spectroscopy using preparation and analysis methods previously reported.^20–22^ Thermogravimetric analysis was used to study thermal stability of the lignin in a nitrogen atmosphere from 100 °C to 800 °C at 10 °C min^−1^ after a drying step at 100 °C for 20 min.

### Blend preparation and characterization

Blends of recycled PET and lignin at 10, 20 and 30 wt% lignin loading of both as-received lignin (L) and thermally treated lignin (L_HT_) were prepared respectively, with 10 wt% of renewable plasticizer relative to the PET weight at 240 °C. The plasticized PET is symbolised as PET_PL_. A Haake MiniLab co-rotating twin-screw extruder (Thermo Scientific) with screw length of 110 mm was used at screw rotation speed of 30 rpm. In a different setup, the extruder was fitted with a die to generate a monofilament of 0.20 to 0.40 mm diameter. The partially renewable blends are identified as PET_PL_/10L, where 10 wt% L was added and PET_PL_/30L_HT_, where 30 wt% L_HT_ were added respectively. All neat PET filaments used in this study were generated at 280 °C and used as reference. A differential scanning calorimeter (DSC Q2000, TA Instruments) was used to determine the thermal transitions of the control PET and its lignin-derived blends. Samples with mass of approximatively 3–4 mg each were loaded in hermetic pans for measurements. A cycle of heating-cooling-heating from −50 °C to 230 °C at 10 °C min^−1^ and an isothermal of 2 min after first heating were used. Thermal decomposition of the blends was evaluated using thermogravimetric analyzer (TGA Q500, TA Instruments) under oxidative atmosphere from 100 °C to 600 °C at 20 °C min^−1^ after a short drying step. A Zeiss EVO MA 15 scanning electron microscope was used to obtain micrographs of the cryo-fractured surfaces of the blends. The samples were kept in 1 M NaOH solution for 20 min at 80 °C after cryofracture before SEM analysis to remove lignin phases from the surface. Washed and dried samples were coated with gold to avoid charging when images were collected. Images were collected at an operating voltage of 20 kV.

Monofilaments of control PET and its lignin-derived blends were tested using an Instron 5943 equipped with Bluehill 3 software and pneumatic side action grip. The crosshead speed was 15 mm min^−1^ and the filaments cross-sectional diameters were used for calculation of cross-sectional area and applied stress. Dynamic mechanical analysis (DMA) measurements were carried out on the monofilaments (diameter 0.20–0.40 mm depending on the sample) at 0.1% strain rate, discrete frequencies of 1, 10, and 100 Hz, and between 30 °C and 150 °C scanned at 3 °C min^−1^. The rheological properties were analyzed using the Discovery Hybrid rheometer (DHR-3, TA instruments). All measurements were carried out in the linear regions at 3% strain in nitrogen atmosphere. Frequency sweeps from 100 to 1 rad s^−1^ at 240 °C and 250 °C were performed.

## Results and discussion

### Lignin structural transformation

Lignin has gained valuable importance recently in preparation of a new class of renewable thermoplastic elastomeric materials.^23,24^ Lignin is an excellent renewable feedstock for manufacturing of environment-friendly materials because of its multifunctional nature and associated chemistries. Here, lignin was thermally treated at 200 °C under vacuum for 60 minutes to avoid thermal decomposition during blending with recycled PET. Detailed insight into the microstructural transformation induced by thermal treatment is important for the final properties of the manufactured blends. ^31^P NMR and 2D HSQC NMR were used to identify and quantify the chemical group profiles of both the as-received and thermally treated lignins.

**Fig. 1:**
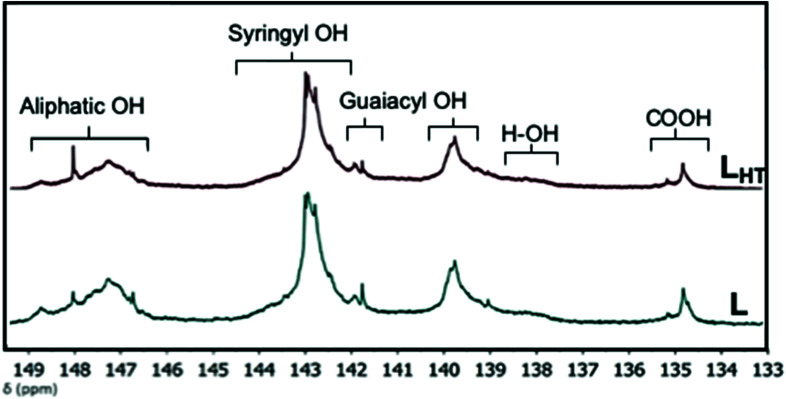
Functional groups identified by quantitative ^31^P NMR measurements after phosphitylation of lignins L and L_HT_.

Phosphitylation of OH groups in lignin allow quantification of different OH moieties of lignin by ^31^P NMR analysis. The ^31^P NMR spectra with chemical shifts and microstructural assignments are shown in Fig. 1, whereas the amount of OH groups determined from the spectra is summarized in Table 1. Thermal treatment decreased the aliphatic OH content of lignin. L has the higher amount of aliphatic hydroxyl (2.01 mmol g^−1^) compared to L_HT_ (1.63 mmol g^−1^). This indicates that during thermal treatment, structural transformation of lignin starts with dehydration which eliminates the side chain OH groups.^25^ The total amount of phenolic OH increased after thermal treatment, however, the carboxylic group content remained the same. L is an organosolv-extracted hardwood lignin and is expected to have phenolic syringyl (S) and guaiacyl (G) hydroxyl groups, with little to no *p*-hydroxyphenyl (H) OH groups.^20,26^ The results are in accordance as these two groups are higher than the H–OH groups. Moreover, thermal treatment led to an overall increase of phenolic OH groups in L_HT_ possibly due to the cleavage of β-O-4 linkages; specifically, increase in H–OH groups signifies additional demethoxylation of S and G-lignin at the 3,5- and 3-positions, respectively.^27^

**Table 1:** Functional groups (mmol g^−1^) of the L lignin and thermally treated lignin (L_HT_) as determined by the quantitative ^31^P NMR method

Samples	Aliphatic OH *δ* 150–145 ppm	Syringyl OH *δ* 144.5–142.25 ppm	Guaiacyl OH		*p*-Hydroxyphenyl OH *δ* 138.5–136.5 ppm	Total phenolic OH *δ* 145–136.5 ppm	Carboxylic group *δ* 136–133.5 ppm
			C^a^*δ* 142.25–141 ppm	NC^b^*δ* 141–138.5 ppm			
L	2.01	2.85	0.80	1.79	0.42	5.86	0.45
L_HT_	1.63	3.08	0.96	2.04	0.56	6.64	0.46

a Condensed.

b Non-condensed. Chemical shifts (*δ*) were ascribed as per Balakshin and Capanema (2015).^20^

**Fig. 2:**
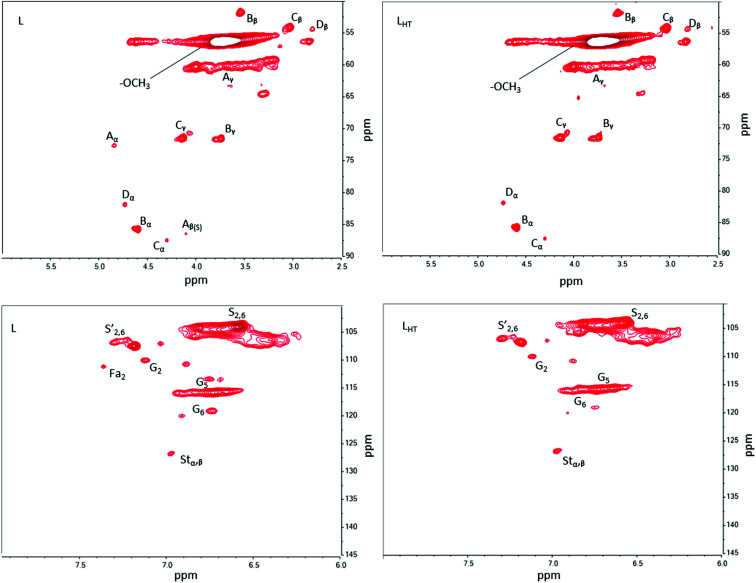
Two-dimensional heteronuclear single quantum coherence (2D-HSQC) NMR spectra of both L and L_HT_ lignins. The top two images are the aliphatic oxygenated side chain region (*δ*_C_/*δ*_H_ 50–90/2.5–6) and the bottom two images represent the aromatic/unsaturated region (*δ*_C_/*δ*_H_ 90–150/6–8).

Two regions of the 2D HSQC NMR spectra analyzed are shown in Fig. 2. Cross peak assignments and corresponding inter-unit linkages are available in Fig. S1 and Table S1 (ESI‡). The regions possess similarities between the two treatments (L or L_HT_) except for a few signals. The cross peaks corresponding to C_α_–H_α_ in β-O-4′ substructures (*δ*_C_/*δ*_H_ = 71.9/4.9) and C_β_–H_β_ in β-O-4 substructures linked to syringyl units (*δ*_C_/*δ*_H_ = 86.3/4.15) were significantly reduced or disappeared after thermal treatment. The C_5_–H_5_ and C_6_–H_6_ in guaiacyl units were also reduced. This indicates that β-O-4′ and β-O-4 aryl ether bonds cleaved during thermal treatment and confirms the ^31^P NMR results discussed above. The β-O-4 linkages are easily altered by heat as discussed in previous reports.^27^ The expectation is that ether radicals and phenoxyl radicals will result from these cleavages and react among themselves to initiate crosslinking reactions (condensation) or remain available for further interactions (physical) during melt blending of lignin with the engineered polyester. Moreover, hindered phenolic OH groups in lignin are free radical scavengers that too are susceptible to these radicals for further condensation reactions. The possibility of condensation reactions occurring among the lignin free radicals generated during thermal treatment explains the increase of 5-substituted groups (S–OH), and condensed G-OH in L_HT_ (Table 1).

Molecular mass and its distribution for both lignins obtained by GPC indicate significant influence of thermal treatment at 200 °C on structural transformation of lignin. GPC results show increases in *M*_n_, *M*_w_ and *M*_w_/*M*_n_ after thermal treatment. L was found to have *M*_n_ = 890, *M*_w_ = 1486, and *M*_w_/*M*_n_ = 1.67, while L_HT_ had *M*_n_ = 1103, *M*_w_ = 1924, and *M*_w_/*M*_n_ = 1.75. The increasd average molecular weight is the result of condensation reactions that occurred during thermal treatment. Evidences of the reactions are found in features detected by ^31^P NMR and 2D HSQC NMR. Thermal behaviors analysis by DSC agrees with these findings as glass transition temperature of lignin increased from 86 °C to 97 °C after thermal treatment (ESI Fig. S2‡). The degree of crosslinking was mild; otherwise, pronounced crosslinking would have enlarged the macromolecules to a higher range. For example, L_HT_ would have had a higher molecular weight and reduced viscosity. The fact that L_HT_ still flows at ∼165 °C is an evidence of mild crosslinking. This also suggests that oxidation was avoided in vacuum. A treatment duration of 60 minutes should have been enough to advance the condensation reactions when conducted in an oxidative atmosphere.^28^

Thermal treatment improved thermal stability of the lignin through removal of volatiles, dehydration, crosslinking in aromatic structures and increasing the degree of condensation.^25^ As-received lignin started to degrade at 185 °C. Thermal treatment shifted the onset of degradation to higher temperatures to accommodate melt mixing with PET at 240 °C. TGA thermograms of L and L_HT_ are shown in Fig. S3 (ESI‡). Weight reduction temperatures recorded at 5% weight loss was 247 °C for L compared to 265 °C for L_HT_. The derivative weight thermogram of as-received lignin has a shoulder from 143 °C to 258 °C that disappeared after thermal treatment by removing low molecular weight volatiles and cleaving thermally liable ether bonds. Overall, thermal treatment under vacuum only changed lignin's structure slightly to improve its thermal stability. This avoids significant oxidative degradation reactions that are detrimental to keeping the lignin malleable. In addition, the soak time of 60 minutes was sufficient to generate lignin that is thermally stable and malleable for blending with engineered polymer matrices.

### Thermal and morphological properties of the compositions

Thermal transition temperatures, calorimetric values, and degree of crystallinity computed from cooling and second heating of DSC thermograms are shown in ESI (Table S3‡). The results suggest that addition of TOFA plasticizer reduces the melting temperature of the neat PET. The melting temperature of PET shifted from 247 °C to 239 °C in presence of 10 wt% TOFA. Plasticizers are small molecular weight materials that are added to help soften the rigid amorphous phase of polymer. It enhances segmental mobility by depressing the glass transition temperature (*T*_g_) of the amorphous phase of the host polymers. The effect of the plasticizer on recrystallization during cooling is observed as the recrystallization temperature shifts from 208 °C to 202 °C.

Addition of lignins (L and L_HT_) in all compositions further reduces the melting temperatures and decreases the heat of fusion. This is evidence for reduction in crystallite sizes in PET with incorporation of lignin in the blends. Also, the difference between the behavior of L series alloys compared to L_HT_ series alloys suggests variance in the degree of interactions between the lignins and PET. In theory, the addition of oligomeric lignin increases the free volume in the PET matrix which leads to the plasticization effect. Additionally, lignin addition shifts the recrystallization temperature (*T*_rec_) of PET to lower temperatures. Shifting of *T*_rec_ and Δ*H*_rec_ suggests that lignin is decelerating the recrystallization and crystal growth during cooling. Conclusions from these results show that interactions exist between both lignins and PET. These interactions could be the hydrogen bonding and π electron interactions. The degree of crystallinity (*χ*_c_) was computed using eqn S1 (ESI‡) and heat of fusion. Presence of plasticizer lowers the crystallinity in PET matrix and presence of lignin further lowers the degree of crystallinity. Lignin loaded (10–30 wt%) PET_PL_ matrix exhibits 17–26% crystallinity.

**Fig. 3:**
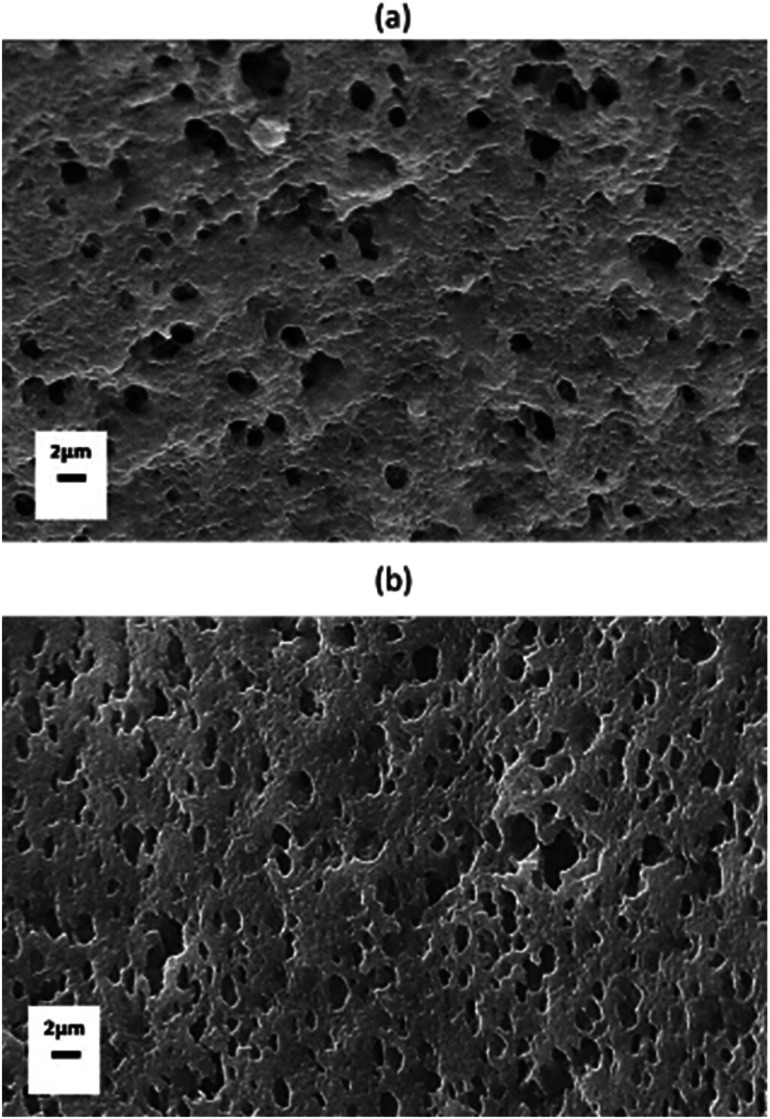
Scanning electron micrographs of cryo-fractured and NaOH etched surfaces of PET_PL_/30L (a) and PET_PL_/30L_HT_ (b) blends.

Microscopic analysis of cryo-fractured surfaces of the blends (Fig. 3) shows that the morphologies depend on the nature of lignin at 30 wt% lignin contents in the blends. The samples were etched in 1 M solution of NaOH to dissolve lignin from the cryo-fractured surface before SEM imaging. In Fig. 3a, the blend of PET and as-received lignin (L) shows lignin as less concentrated but larger droplets in the PET matrix. Lignin droplet sizes vary from 1 to 2 micrometers. However, the thermally treated lignin-derived PET blend shows formation of homogenously dispersed cavities after removal of lignin macromolecules (0.2 to 2 micrometer). Controlling lignin–lignin intermolecular interaction through thermal treatment by decreasing aliphatic hydroxyl helps avoid coalescence of the lignin phase during mixing in the engineered polyester matrix. It has been reported that controlling microstructure and dispersion of lignin in thermoplastic blends is important for improved performance.^29^

### Interfacial interactions-performance relationships

#### Mechanical properties

**Fig. 4:**
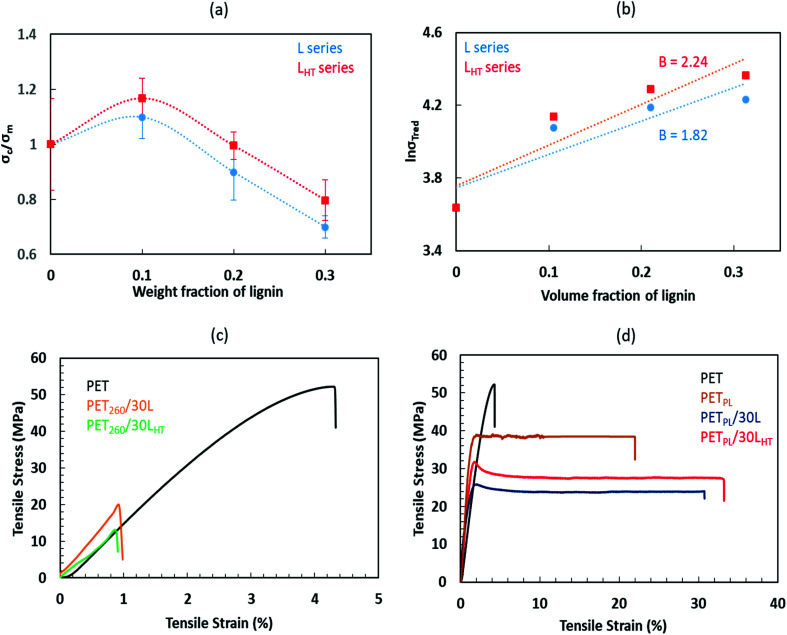
Ratios of tensile strength of lignin-loaded matrices over those of neat PET at different lignin weight fraction (a), the natural logarithm of reduced tensile strength as a function of volume fraction of lignin (b), Tensile stress–strain curves of (c) neat PET and its lignin-derived blends amalgamated at 260 °C and (d) plasticized PET and its lignin-derived blends amalgamated at 240 °C showing enhanced mechanical properties of the blends due to plasticization effects of tall oil fatty acid and thermal treatment of lignin.

Blending of lignin in thermoplastics often reduces tensile strength. This is primarily because the lignin forms large domains in thermoplastic matrices causing defect centers. However, lignin was reported to impart rigidity and stiffness in some systems.^23^ Improving overall performance of the blends relies on the level of interactions between the lignin and the host polymer molecules. Fig. 4a illustrates the relationship between the tensile strength presented as a ratio of tensile strength of the lignin-derived composites over the tensile strength of the matrix, in this case plasticized PET, as a function of weight fraction of lignin in the blends. The (*σ*_c_/*σ*_m_) increases at low lignin loadings then diminishes with increasing lignin amount. Obviously, weak interactions between the PET and lignin generate large lignin domains in the blends (Fig. 3a) that affect performance of the blends negatively. Also, there is a possibility of thermal degradation of lignin during mixing at 240 °C leading to inferior performance. In this study, we find that the thermal pre-treatment improves lignin stability and helps to improve lignin dispersion in the PET matrix and, thus, the mechanical properties. Although thermal treatment increases the molar mass of L_HT_, mechanical shear during blending helps to break the aggregates of lignin macromolecules into finer droplets compared to the system consisting of original lignin, L. Low lignin-loaded compositions show slightly improved performance of the plasticized PET matrix when combined with L_HT_ as compared to its control counterparts.

The compositional dependence of the mechanical properties limits our ability to draw a definitive conclusion on the interactions in each blend. We observe, however, that better interactions exist between the L_HT_ and the PET compared to L blends. Nevertheless, the results of L series imply that some level of interactions is also occurring between L and PET, possibly competing hydrogen bonding between the lignin OH and PET end groups (ester and ethylene groups) and π–π interaction between aromatic groups of lignin and PET. Overall, the representative tensile stress–strain curves of the PET and its high lignin containing blends formulated at 260 °C and 240 °C (respective Fig. 4c and d) are in agreement that a combination of plasticization effects induced by the addition of TOFA and thermal treatment of lignin helped to enhanced performance of the blends. It may also be noted that the presence of lignin in the TOFA modified PET matrix enhances ductility of the product significantly. Recycled PET-based lignin-derivatives with 30% or higher elongation at failure is significant compared to the neat waste PET and plasticized waste PET that shows <5% and ∼20% elongation, respectively.

Fig. 4b shows natural logarithm of reduced tensile strength as a function of volume fraction of lignin. The reduced tensile strength is described by eqn (1).^30^ The plot is used for quantitative estimation of interaction using the composition dependence of strength model. The model relates the interfacial interactions, structure and the mechanical properties of the blends. It is expressed to reflect the effect of volume fraction (*φ*) of the dispersed component, and the load bearing capacity of the dispersed lignin constituent (*B*), which is dependent on interfacial adhesion.^31,32^1
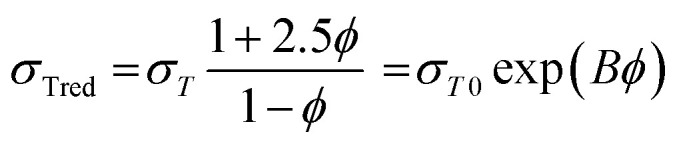
here, *σ*_Tred_ is the reduced tensile strength of the blend, *σ*_T_ and *σ*_T0_ are the tensile strength of the blends and the matrix, respectively.

The results are summarized in Table 2. It reveals that parameter *B*, which is the slope of the linear correlation applied to the data, increased from 1.82 to 2.24 when the thermally treated lignin L_HT_ was used instead of L highlighting divergent interfacial adhesion. Thermal treatment was beneficial to improve thermal stability, control lignin–lignin intermolecular interactions and to control lignin-PET interaction likely through a combination of hydrogen bonding and π electron interactions that is clearly different in the composites based on as-received lignin (L). Calculated tensile stress of the matrix (*σ*_T0_) for both cases (L and L_HT_ series) agrees well with the measured value.

**Table 2:** Quantitative estimation of interactions computed from mechanical properties of the blends

Lignin	Treatment	*σ*_T0_ (MPa)		*B*	*R* ^2^
		Measured	Calculated^b^		
L	As-received	38.12 (6.31)^a^	42.09	1.82	0.87
L_HT_	Heat treated		42.94	2.24	0.89

a standard deviation is shown in parenthesis.

b Computed from the *y*-intercept of ln *σ*_Tred_*vs.* volume fraction of lignin plots.

#### Dynamic mechanical analysis

**Fig. 5:**
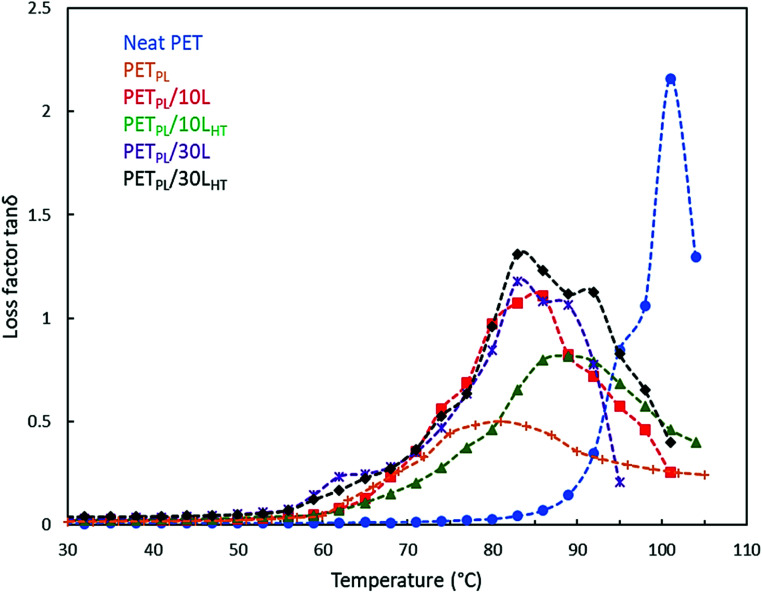
Loss tangent peak of PET, plasticized PET and its derived blends with L and LHT at in different compositions at 10 Hz frequency.

Loss tangent (tan *δ*) peaks for PET and its lignin-based alloys at 10 Hz frequency are shown in Fig. 5. Neat PET shows a narrow tan *δ* peak representative of its glass transition temperature (*T*_g_) at 101 °C. This shift is likely attributed to the presence of TOFA at 10 wt% of PET in each blend. The tan *δ* peaks became broader in the presence of lignin in PET matrix and increasing lignin content increased the *T*_g_. When lignin L is used, moderate interactions with PET matrix are expected. The *T*_g_ is 86 °C for a 10 wt% lignin loading in plasticized PET; use of L_HT_ makes better dispersion and interactions with PET matrix and thus, a slight increase in *T*_g_ is observed (89 °C). Similar observations were made in the case of higher lignin loading (30 wt%) in blends.

The loss tangent data represents the energy dissipated by the materials under cyclic load. Application of the Arrhenius equation to the loss factor (tan *δ*) peak temperature as a function of frequency data provides quantitative evaluation for the relaxation behavior of PET phase in the blends. In this instance, the Arrhenius equation can be expressed in the following form:2
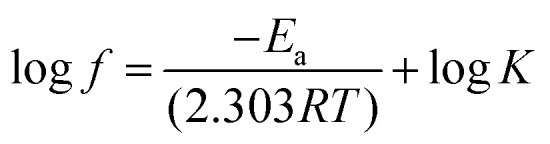
here *T* is the absolute temperature at which the loss maximum is observed at frequency *f*, *R* is the gas constant, *K* is an arbitrary constant, and *E*_a_ is activation energy associated with glassy to rubber transition or relaxation. Table 3 shows computed *E*_a_ data for neat PET (404 kJ mol^−1^). Addition of TOFA reduced the activation energy in PET. However, the thermally treated lignin alloys have higher *E*_a_ compared to the as-received lignin compositions in TOFA plasticized PET; although, the increase in *E*_a_ becomes marginal at high L_HT_ content in the blend. Two phenomena are occurring simultaneously. First, the plasticizer is helping to depress *T*_g_ while rigid lignin hinders segmental motion of PET. Treated lignin L_HT_ has a higher degree of interaction with PET matrix and thus restrains the flexibility of the PET phase. At high L_HT_ loading, however, the advantageous effect of improved dispersion on relaxation of PET matrix diminishes.

**Table 3:** Temperatures corresponding to the loss tangent peak (*T*_g_) at different frequencies from the dynamic mechanical analysis, and the activation energy (*E*_a_) associated with thermal relaxation at *T*_g_

log *f* (Hz)	Neat PET		PET_PL_		PET_PL_/10L		PET_PL_/10L_HT_		PET_PL_/30L		PET_PL_/30L_HT_	
	*T*_g_ (°C)	*E*_a_ (kJ mol^−1^)	*T*_g_ (°C)	*E*_a_ (kJ mol^−1^)	*T*_g_ (°C)	*E*_a_ (kJ mol^−1^)	*T*_g_ (°C)	*E*_a_ (kJ mol^−1^)	*T*_g_ (°C)	*E*_a_ (kJ mol^−1^)	*T*_g_ (°C)	*E*_a_ (kJ mol^−1^)
0	92		70		71		74		80		83	
1	101	404	81	270	86	180	89	200	98	195	101	198
2	104		87		98		98		104		107	

### Process engineering and degradation parameters of partially renewable blends

PET is a semi-crystalline polymer. Its normal processing temperatures is between 270 °C to 280 °C. Blending of lignin with PET requires manipulating the PET thermal behavior to prevent degradation of lignin. Our approach to address this involves use of a renewable plasticizer to soften PET matrix. In practice, low molecular weight plasticizers are often added to increase the flexibility at room temperature and to improve processing. All blends studied in this report were mixed at 240 °C, a processing temperature of PET that was enabled by the addition of plasticizer.

In thermoplastic matrix-lignin systems, compatibility and dispersion of lignin are desired for enhanced mechanical properties.^33^ Often, partial or full miscibility help improve the properties of the blends. Our results show some level of affinity between the L lignin and PET. However, such interactions are improved when L_HT_ is used. Miscibility could have been increased by raising the melt-mixing temperature, but that approach would degrade the lignin, causing charring and subsequent phase separation during shear mixing. Viscous heating is another cause of lignin degradation during melt-mixing. Thus, rheological behaviors of the components and the blends are important. Ultimately, the process depends on the molecular structures of the components. Therefore, differences in lignin molecular structure are expected to affect rheological behaviors of the resulting polymer blends.

**Fig. 6:**
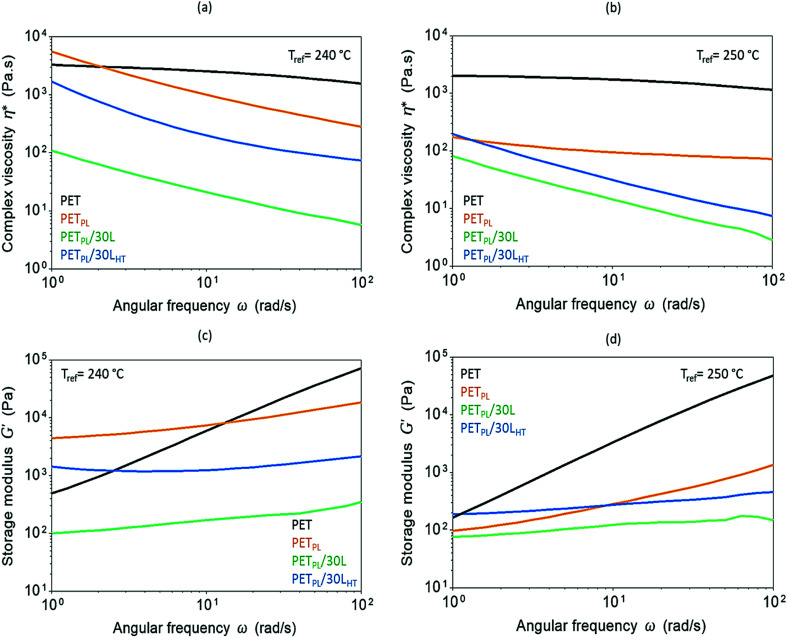
Frequency-dependent complex viscosity (η*) at *T*_ref_ = 240 °C (a) and 250 °C (b) and frequency-dependent storage modulus (*G*′) at *T*_ref_ = 240 °C (c) and 250 °C (d) of recycled PET, its plasticized resin (PET_PL_), and its lignin-derived blends (PET_PL_/30L and PET_PL_/30L_HT_).

Influence of lignin molar structure on flow characteristics of the PET blends is illustrated in Fig. 6. The angular frequency (*ω*) dependence of the complex viscosity (η*) and the storage modulus (*G*′) were used to study flow characteristics of neat PET, its plasticized blend at 10 wt% plasticizer amount (PET_PL_), and its lignin derived blends at high-lignin-loading (30 wt%) at reference temperatures of 240 °C and 250 °C. Plasticization outcome is clear as the viscosity decreased at both temperatures with increasing frequency. The materials stiffness at 240 °C is higher compared to its stiffness at 250 °C (Fig. 6c and d). Addition of lignin further decreases the viscosity and the storage modulus at both reference temperatures, suggesting a role of viscous oligomeric lignin on plasticization of the PET. Interestingly, the blend with thermally treated lignin (PET_PL_/30L_HT_) has higher viscosity and storage modulus than the as-received lignin blend (PET_PL_/30L). As discussed earlier, this is due to the homogenous dispersion of L_HT_ in PET (as shown by microscopy), and possible enhanced interfacial interactions through combination of hydrogen bonding and π–π interaction of lignin with PET chains and restrained chain disentanglement along with retardation of segmental relaxation (in accordance to DMA data around the glass transition temperature *T*_g_ of the blends).

Thermogravimetric analysis was used to evaluate thermal degradation behavior of the blends in oxidative atmosphere. Mass loss data collected at 20 °C min^−1^ scanning rate are shown in Fig. S4 in ESI.‡ The results are summarized in Table 4. Addition of lignin reduces the temperature corresponding to 5% mass loss (*T*_i_) and the onset temperature (*T*_d_) but increases the derivative weight peak temperature. Addition of lignin improves net degradation of the blends and confirms the effect of thermal treatment of lignin on the thermal stability of the blends. L_HT_ blend is marginally more stable at higher temperatures than the L blend. Additionally, mass at 500 °C increased with the addition of lignin showing the protective effect of lignin at higher temperatures.

**Table 4:** Thermal degradation parameters of neat PET, PET_PL_/30L and PET_PL_/30L_HT_

	PET	PET_PL_/30L	PET_PL_/30L_HT_
5% weight loss temp. *T*_i_ (°C)	391	290	303
Onset temperature *T*_d_ (°C)	400	388	390
DTG peak temperature (°C)	436	438	440
Mass at 300 °C (%)	99.9	93.9	95.3
Mass at 500 °C (%)	14.3	27.1	27.6

## Conclusion

We have successfully demonstrated that lignin dispersion and interfacial interaction can be controlled in recycled PET/lignin alloys through thermal pre-treatment of lignin. The addition of renewable plasticizer at 10 wt% relative to PET helped to soften PET below its normal processing temperature to avoid further degradation of lignin during mixing. Thermal treatment of lignin decreases the aliphatic hydroxyl group, minimizes lignin–lignin intermolecular interactions and improves lignin thermal stability. Relative tensile failure stress of lignin-PET alloys with respect to that of the PET matrix (*σ*_c_/*σ*_m_) improves by 15%, in the presence of thermally pretreated lignin at a composition of 30 wt%. Small amount of lignin (10%) in the PET matrix shows a reinforcing effect. Computed interfacial interaction of the dispersed lignin with the PET matrix improves significantly when thermally pretreated lignin is used. This clearly shows that combined interactions (hydrogen bonding and π electron interactions) are enhanced after heat treatment of lignin. Dynamic mechanical analysis and rheology study confirm balanced interactions between the PET and heat-treated lignin as oligomeric lignin is known to enhance chain disentanglement (shear thinning) and restrain segmental motion resulting in increased *T*_g_. Our formulations use lignin, a low-cost renewable resource, post-industrial PET waste destined for landfills, and renewable plasticizer TOFA, a low-priced by-product from pulping industries to develop a renewable material with well dispersed lignin domain and good mechanical performance. This work highlights development of renewable thermoplastics based on lignin and sustainable industrial waste PET, offering a path for high-volume utilization of lignin in a value-added form.

## Conflicts of interest

There are no conflicts to declare.

## Supplementary Material

RA-009-C9RA07052D-s001
