# Supplementary material for: A tough and sustainable fiber-forming material from lignin and waste poly(ethylene terephthalate)
Source: RSC Adv. 2019 Oct 1;9(54):31202–11. doi: 10.1039/c9ra07052d (PMC9072396; doi:10.1039/c9ra07052d)
Supplement: RA-009-C9RA07052D-s001 [file RA-009-C9RA07052D-s001.pdf]

Electronic Supplementary Material (ESI) for RSC Advances.
This journal is © The Royal Society of Chemistry 2019

SUPPORTING INFORMATION

A tough and sustainable fiber-forming material from lignin and waste poly(ethylene terephthalate)

Kokouvi M. Akato,[†,‡] Ngoc A. Nguyen,[§] Kalavathy Rajan,[†] David P. Harper,[†] Amit K. Naskar*,[†,‡,§]

[†]Center for Renewable Carbon, University of Tennessee, Knoxville, Tennessee 37996, United States

[‡]Bredesen Center for Interdisciplinary Research and Graduate Education, University of Tennessee, Knoxville, Tennessee 37996, United States

[§]Carbon and Composites Group, Chemical Sciences Division, Oak Ridge National Laboratory, Oak Ridge, Tennessee 37831, United States

*Corresponding author: naskarak@ornl.gov

Detailed GPC Analysis

GPC was performed for both L and L_{HT} lignins using a Tosoh EcoSEC gel permeation chromatography (GPC) system with a refractive index (RI) detector equipped with a flow reference cell. Prior to measurements, lignin was dissolved in THF at a concentration of 1 mg/mL and filtered using a 0.22 µm membrane. The instrument and reference cell flow rates were set to 0.35 mL/min and the analysis was performed at 40 °C. Sample injections of 10 µL were separated using two consecutive Tosoh TSKgel SuperMultiporeHZ-M analytical columns (4.6 mm I.D., 150 mm length, 5 µm particle size) and a TSKgel SuperMultiporeHZ-M guard column using a total run time of 15 min. Evaluation of the number-average molecular weight (M_n), weight-average molecular weight (M_w) and their ratio (PDI) was complete using in-house polystyrene standard curves in the range of 600-7.5×10^6 Da.

^{31}PMNR

Table S1 Assignment of hydroxyl groups peaks in ^{31}P NMR spectroscopy.

Sample name/ mmol per g lignin	Assignments, δ (ppm)
Aliphatic OH	150-146
S-OH condensed	144.5-143.5
S-OH non-condensed	143.5-142.25
G-OH condensed	142.25-141
G-OH non-condensed	141-138.5
H-OH	138.5-136.5
COOH	136-133.5

2D ^{1}H–^{13}C HSQC NMR

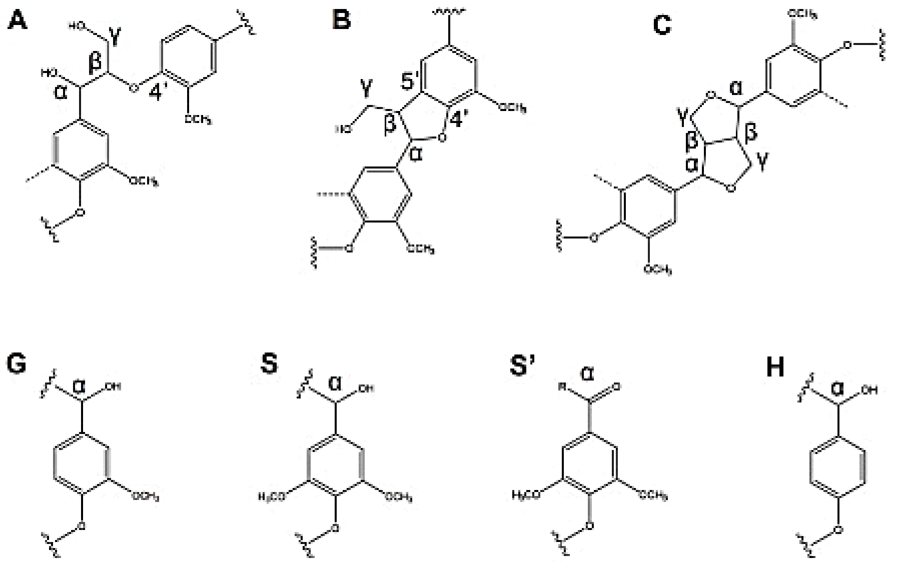

Figure S1. Lignin substructures detected by 2D HSQC NMR. (A) β-O-4'; (B) β-5' (phenylcoumaran structure); (C) β-β' (resinol structures); (G) guaiacylpropane unit; (S) syringyl propane unit; (S') syringyl propane unit with carbonyl at Cα; (H) p-hydroxyphenolpropane unit [1].

Table S2. ^{13}C and ^{1}H assignments of the lignin signals in 2D HSQC spectra [2].

Label	δC/δH (ppm)	Assignment
B_β	53.1/3.4	C_β–H_β in phenylcoumaran substructures (B)
C_β	53.5/3.1	C_β–H_β in β–β′ resinol substructures (C)
–OCH3	55.6/3.73	C–H in methoxyls
A_γ	59.4/3.4 and 3.7	C_γ–H_γ in γ– hydroxylated β-O- 4′ substructures (A)
I_γ	61/4.1	C_γ–H_γ in cinnamyl alcohol end-groups (I)
B_γ	63.4/3.6	C_γ–H_γ in phenylcoumaran substructures (B)
Hk_γ	67.5/4.2	C_γ–H_γ in Hibbert ketone structuresb
C_γ	71.2/4.2	C_γ–H_γ in β–β′ resinol substructures (C)[b]
A_α	71.9/4.9	C_α–H_α in β-O-4′ substructures (A)
X_2	73/3.1	C_2–H_2 in xylan substructures (X)
X_3	74/3.3	C_3–H_3 in xylan substructures (X)
X_4	75.7/3.5	C_4–H_4 in xylan substructures (X)
A_β	80.4/4.5,<br>84.4/4.4<br>and 85.6/4.2	C_β–H_β in β-O-4′ substructures (A)
$A_{ox}\beta$	83/5.2	C_β–H_β in α-oxidized β-O-4′ substructures (Aox)
C_α	85.5/4.6	C_α–H_α in β–β′ resinol substructures (C)
B_α	87.7/5.5	C_α–H_α in phenylcoumaran substructures (B)
T_8	94.4/6.6	C_8–H_8 in tricin units (T)
T_6	99.5/66.2	C_6–H_6 in tricin units (T)
$T_{2,6}$	104.5/7.4	C_2–H_2 and C6-H6 in tricin units (T)
$S_{2,6}$	104.2/6.7	C_2–H_2 and C6–H6 in syringyl units (S)
T_3	107/7.2	C_3–H_3 in tricin units (T)
$S'_{2,6}$	107.4/7.4	C_2–H_2 and C6–H6 in syringyl units with α oxidization(S′)
G_2	110.2/6.9	C_2–H_2 in guaiacyl units (G)
Fa_2	111.5/7.3	C_2–H_2 in ferulate (Fa)
G_5/G_6	115/6.7 and<br>119.7/6.8	C_5–H_5 and C6–H6 in guaiacyl units (G)
Fa_6	123.1/7.1	C_6–H_6 in ferulate (Fa)
HMF	123.6/7.5	C_3– H_3 in 5-O-substituted furfurals -like units
$St_{\alpha,\beta}$	126.6/6.9	C_α–H_α and C_β–H_β in stilbene structures (St)
$H_{2,6}$	128.2/7.2	$C_{2,6}$–$H_{2,6}$ in p-hydroxyphenyl units (H)
I_α	130.6/6.3	C_α–H_α in cinnamyl alcohol end-groups (I)
$Pca_{2,6}$	130.1/7.5	C_2–H_2 and C_6–H_6 in p-coumarate (Pca)
$Pb_{2,6}$	131.6/7.7	C_2–H_2 and C_6–H_6 in p-benzoate (Pb)
HMF	179/9.6	C_α–H_α in 5-O-substituted furfurals -like units

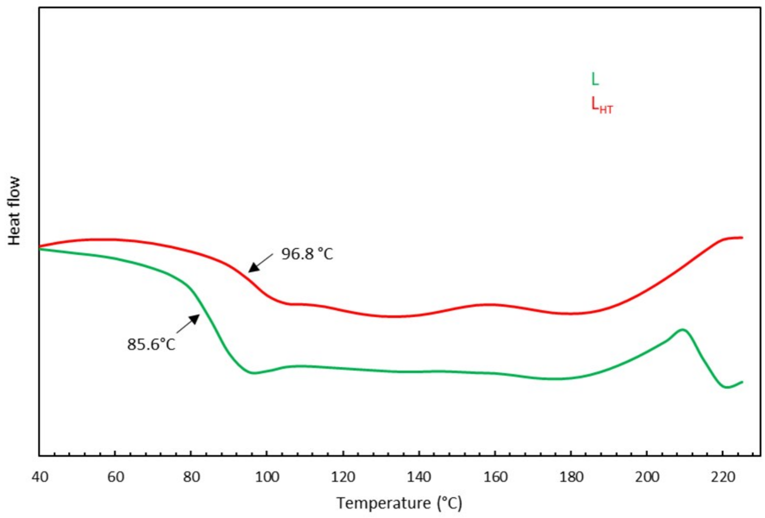

Figure S2 DSC thermograms of L and L_{HT} in nitrogen atmosphere showing the glass transition temperatures (T_g).

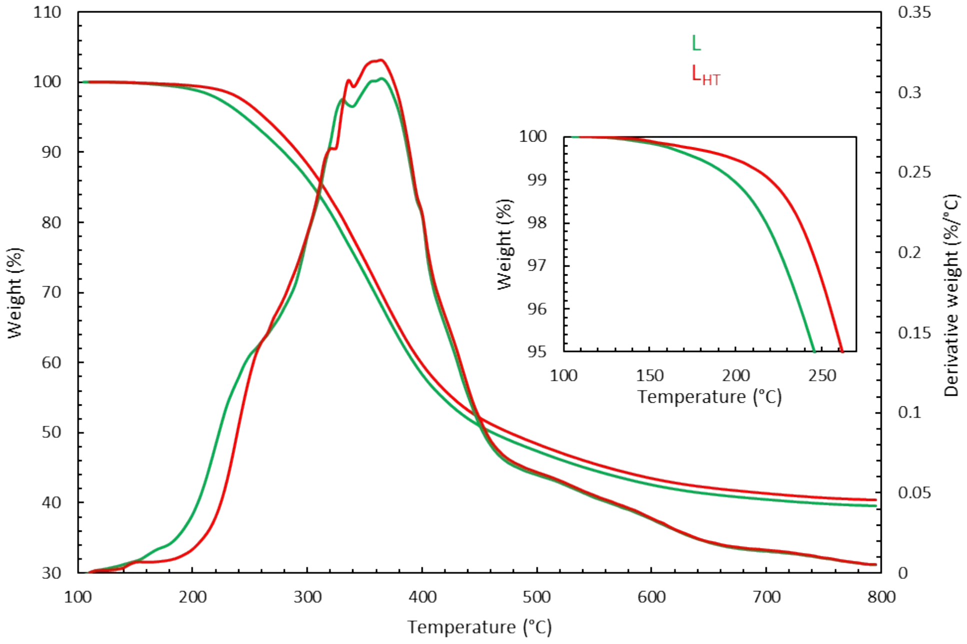

Figure S3. TGA and derivative weight thermograms of L and L_{HT} in nitrogen atmosphere showing the effect of thermal treatment on lignin thermal stability.

Table S3 Thermal behavior temperatures, calorimetric values, and degree of crystallinity of PET and its lignin derived blends.

Samples	T_m (°C) [a]	ΔH_m (J/g) [a]	T_{rec} (°C)	ΔH_{rec} (J/g)	X_c (%) [b]	X_c (%) [a]
PET	247	46	208	53	57	33
PET_{PL}	239	41	202	49	23	29
PET_{PL}/10L	237	33	209	44	23	24
PET_{PL}/20L	232	32	203	39	31	23
PET_{PL}/30L	229	29	198	33	30	21
PET_{PL}/10L_{HT}	236	36	208	41	30	26
PET_{PL}/20L_{HT}	233	29	203	38	23	21
PET_{PL}/30L_{HT}	231	24	199	32	20	17

[a] Values obtained from second heating curves [b] Computed using first heating curves

The degree of crystallinity (χ_c) was computed using the first heating curves information and applying the following equation.

$$\chi_c = \frac{\Delta H_m}{W_f \times \Delta H_{100}} \times 100\% \qquad \text{(S1)}$$

where ΔH_m is the melting enthalpy from the first heating curve, W_f is the PET weight fraction in each composition and ΔH_{100} is the theoretical fusion enthalpy of 100% crystalline PET (140 J/g) [3]

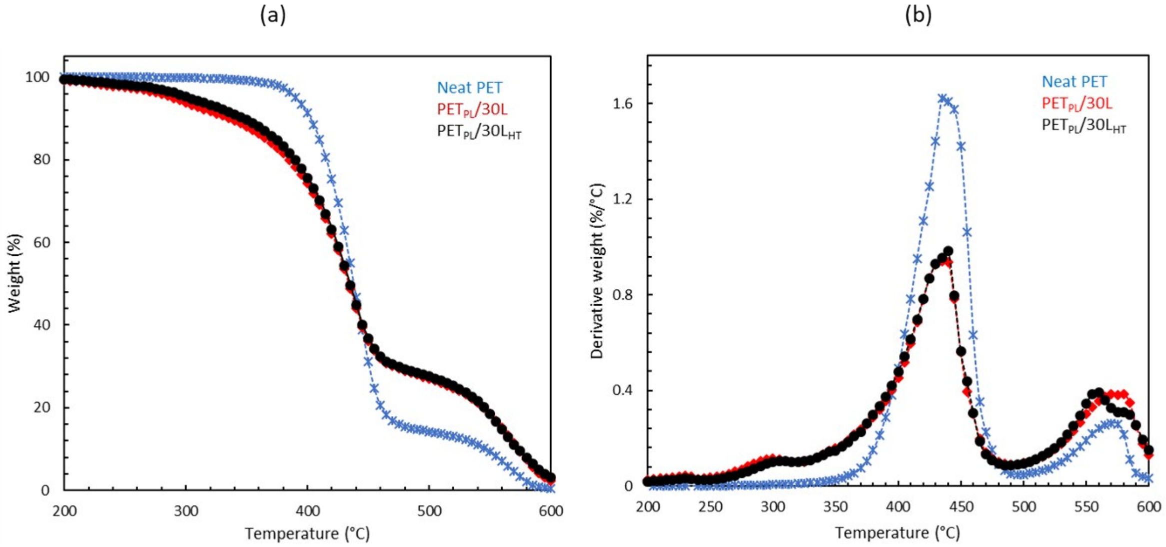

Figure S4. Thermal decomposition of PET and its lignin derived blends at 30 wt.% lignin contents in oxidative atmosphere at 20°C/min.

1. Nguyen, N.A., et al., *An Acrylonitrile–Butadiene–Lignin Renewable Skin with Programmable and Switchable Electrical Conductivity for Stress/Strain-Sensing Applications.* Macromolecules, 2018. **51**(1): p. 115-127.
2. Constant, S., et al., *New insights into the structure and composition of technical lignins: a comparative characterisation study.* Green Chemistry, 2016. **18**(9): p. 2651-2665.
3. Wunderlich, B. Chapter 2 - The basis of thermal analysis. In Thermal Analysis, Wunderlich, B., Ed.; Academic Press: 1990; pp 1-36.
